# Association of hardware removal with secondary osteonecrosis following femoral neck fractures: a systematic review and meta-analysis

**DOI:** 10.1186/s13018-023-04427-8

**Published:** 2023-12-06

**Authors:** Qilong Jiang, Yu Deng, Yang Liu, Zhi Zhao, Yu Chen, Xinwen Bai, Hao Hong

**Affiliations:** Department of Orthopaedic Surgery, Chongqing Orthopedic Hospital of Traditional Medicine, No. 9, Jiefang West Road, Chongqing, 400010 China

**Keywords:** Femoral neck fracture, Hardware removal, Internal fixation, Meta-analysis, Osteonecrosis of femoral head

## Abstract

**Background:**

It has been controversial that whether hardware removal will increase the risk of osteonecrosis of femoral head (ONFH) in fracture-healed patients who underwent internal fixation for femoral neck fractures (FNFs). This meta-analysis aimed to clarify the association of hardware removal with secondary hardware removal-induced ONFH (HR-ONFH).

**Methods:**

Four electronic databases (PubMed, Embase, Web of Science, Cochrane Library) were searched for eligible studies published up to March 10, 2023. Studies reporting the relative risk of hardware status (i.e., risk rate, odds ratio [OR], or hazard ratio [HR]) were included. Newcastle–Ottawa scale (NOS) was used to assess risk of bias of included observational studies. Review Manager software was used to pool ORs and adjusted ORs.

**Results:**

Five studies were included into quantitative synthesis. Hardware removal was associated with a reduced risk of HR-ONFH in the synthesis of crude odds ratios (OR, 0.62, 95% CI 0.39–0.96). In the synthesis of adjusted odds ratios, hardware removal was associated with an increased risk of HR-ONFH (OR, 1.76, 95% CI 1.23–2.51).

**Conclusion:**

This study demonstrates that hardware removal was associated with an increased incidence of HR-ONFH in fracture-healed patients who underwent internal fixation due to FNFs.

## Introduction

Internal fixation has been performed frequently to treat femoral neck fractures (FNFs) [1]. Generally, hardware removal surgery is not necessary after femoral neck fracture healing. However, due to cultural and religious diversity in regions, a high rate of hardware removal surgery has been reported [2, 3]. Despite additional health and economic burden, the occurrence of sequential osteonecrosis of femoral head (ONFH) has been stated to be associated with prior hardware removal surgery [4]. Such hardware removal-induced ONFH (HR-ONFH) may lead to devastating consequences. Only from a medical perspective, HR-ONFH is preventable by renunciation of hardware removal surgery. To date, the majority of published studies have focused on predictors of ONFH prior to hardware removal surgery [4–9]. There are only a few studies assessing the association between hardware removal and sequential HR-ONFH. More than that, the conclusions regarding this topic were controversial across the literature, and no relevant systematic review could be retrieved from mainstream databases [2, 5, 9, 10]. Whereas we conducted this systematic review and meta-analysis, aiming to clarify the association of hardware removal with secondary HR-ONFH in bone-healed patients who underwent internal fixation for FNFs, further to provide evidence-based information to help surgeons and patients make an informed decision regarding internal hardware removal surgery.

## Materials and methods

The Preferred Reporting Items for Systematic Review and Meta-Analysis (PRISMA) guidelines were used to conduct and report the present study [11].

### Search strategy

We conducted the search of four electronic databases (PubMed, Embase, Web of Science, Cochrane Library) from the inception date up to March 10, 2023. An updated search was performed on March 31, 2023. The vocabulary and syntax were tailored precisely to the database. The first search contained variants of title/abstract/keywords and medical topic heading phrases, such as ("femoral neck fractures" OR "femur neck fractures" OR "fractures of femoral neck" OR "fractures of femur neck") AND ("avascular necrosis of the femoral head" OR "osteonecrosis" OR "necrosis"), which were changed by the different databases as necessary. Publication language was restricted by English. The article screening was independently carried out by two authors. Consensus of selection was reached by discussion with a third author. Additional references screening of included studies was performed for relevant eligible articles.

### Inclusion and exclusion criteria

Inclusion criteria were as follows: (1) cohort studies or case–control studies; (2) hardware removal was analyzed as an independent variate; (3) relative risk estimate of hardware status (i.e., risk rate [RR], odds ratio [OR], or hazard ratio [HR]) was reported. Exclusion criteria were as follows: (1) ONFH occurred prior to hardware removal surgery, which could be identified by radiography before removal surgery; (2) follow-up duration after hardware removal was shorter than three months.

### Data extraction

Predesigned data extraction form was used to take data extraction by two independent surgeons. Any discrepancy was resolved by discussion with a third author. Extracted data involve: author, publication year, study design, number of patients, age, hardware type, follow-up duration, adjusted covariates, outcome parameters (e.g., RR, OR, or HR).

### Assessment of study quality

We utilized Newcastle–Ottawa scale (NOS) to assess risk of bias of included observational studies. The assessment is comprised of eight items categorized into three groups (the selection of the study groups, the comparability of the groups, and the ascertainment of the outcome). The total score ranged from 0 (lowest quality) to 9 points (highest quality). Each study was evaluated by two surgeons independently. Disagreement was resolved by a third author.

### Statistical analysis

Review Manager software version 5.4.1 (Nordic Cochrane Centre, Cochrane Collaboration, Denmark) was used to conduct the meta-analysis. Both crude odds ratio and adjusted odds ratio were taken into meta-analysis. OR and 95% confidence interval (CI) were converted into log [OR] and stander error (SE), respectively. Generic inverse variance method was utilized for data synthesis. Fixed-effect model was used for all outcome parameters due to small study size [12]. Statistical heterogeneity was tested by *I*^2^. *I*^2^ lower than 50% was considered low heterogeneity. *I*^2^ between 50 and 75% was considered moderate heterogeneity, and *I*^2^ greater than 75% was considered significant heterogeneity. For composite with moderate or significant heterogeneity, we executed a sensitivity analysis by removing study with small sample size. Forest plots were generated to provide visual view of analyzed outcomes. All estimates were presented with a 95% CI. A *p* value less than 0.05 was considered to indicate statistically significant difference.

## Results

### Literature search results and study characteristics

**Fig. 1:**
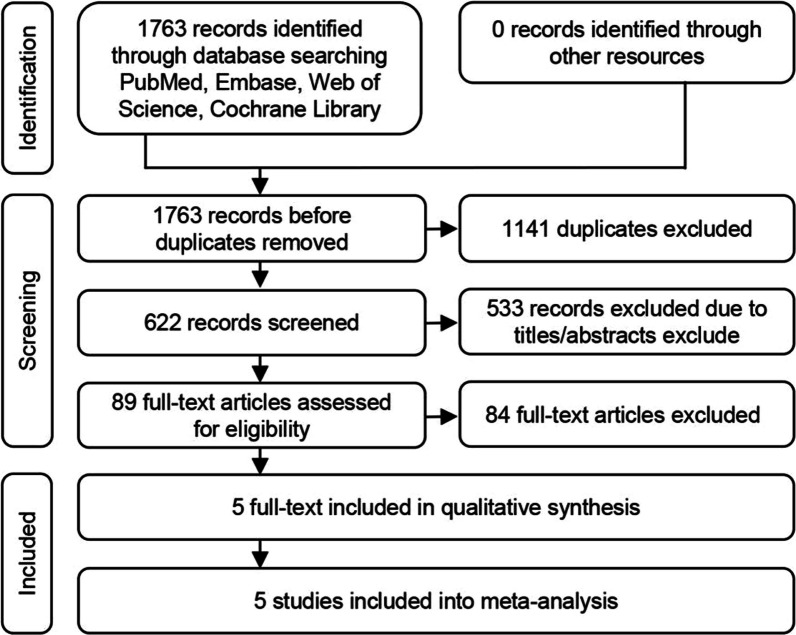
PRISMA 2020 flow diagram for systematic reviews

A total of 1763 records were identified through aforementioned four electronic databases. 1141 duplicates were removed with the application of Endnote tool. 622 studies were excluded following screening of title and abstracts. After 533 records excluded, 89 full-text were assessed for eligibility. Finally, five studies were included into quantitative synthesis [2, 5, 9, 10, 13]. The search flowchart is shown in Fig. 1.

The publication date of included articles ranged from November 2012 to June 2020. Totally, 747 patients with femoral neck fractures were treated with internal hardware. The interval of age ranges from 18 to 85 years. Follow-up duration was between 9 months and 15 years. Events of hardware removal surgery were reported (103/650, 15.85%) in four studies [2, 5, 9, 10]. The reasons for undergoing hardware removal surgery involved slight symptoms (e.g., thigh pain) and patient’s preference. In the whole five studies, multivariate logistic regression analysis was carried out to adjust confounding factors. The Characteristics of included studies have been shown in Table 1.

**Table 1:** Characteristics of included studies

	Author	year	Study design	No. of patients	Age range (years)	Implant type	Follow-up (months)	Adjusted covariates
1	Ai et al. [2]	2012	Retrospective	99	45–85	Cannulated screws	28–60	Age, displacement of fractures, quality of reduction, removal of implants
2	Chang et al. [13]	2019	Case control	102	50–60	Internal fixation without restriction	12–16	Reduction quality, emergency room to operating room (< 6 h or ≧ 6 h), removal of implant
3	Pei et al. [9]	2020	Retrospective	250	18–60	Hollow compression screws	12–180	Type of fracture, the quality of reduction, the removal of internal fixation, BMI, and ASA classification
4	Wang C et al. [10]	2015	Retrospective	150	20–80	Cannulated screws	34–41	Garden classification, displacement of center of femoral head, displacement of deepest of femoral head foveae, rotational displacement
5	Wang T et al. [5]	2014	Retrospective	146	18–68	Cannulated cancellous screws	84	Garden classification, reduction quality, preoperative traction, and implant removal

### Risk of bias

ALL included studies had a NOS score ranging from 7 up to 9, indicating high methodological quality. The detailed assessment has been shown in Table 2.

**Table 2:** Quality assessment of each included study by the Newcastle–Ottawa scale (NOS)

Study	Selection				Comparability	Outcome measurement		Statistical analysis		Total score
	Patient definition^a^	Representativeness of patients^b^	Selection of controls^c^	Definition of controls^d^	Control for important factor^e^	Reliability of outcome measure^f^	Validity of outcome measure^g^	Sample size^h^	Statistical method^i^	
Ai et al. [2]	1	1	1	1	1	1	1	0	1	8
Chang et al. [13]	1	0	1	1	0	1	1	1	1	7
Pei et al. [9]	1	1	1	1	0	1	1	1	1	8
Wang C et al. [10]	1	1	1	1	1	1	1	1	1	9
Wang T et al. [5]	1	1	0	1	1	1	1	1	1	8

^a^The inclusion/exclusion criteria are clearly defined

^b^How were cases selected? (e.g., random sample)

^c^Controls were derived from the same community as patients

^d^Controls defined as individuals without avascular necrosis of the femoral head

^e^Confounding factors have been well adjusted

^f^The measure of avascular necrosis of the femoral head has documented reliability

^g^The measure of avascular necrosis of the femoral head has documented validity

^h^The sample size was justifed

^i^The statistical analysis was clearly presented and was appropriate

### Synthesis of crude odds ratios

**Fig. 2:**
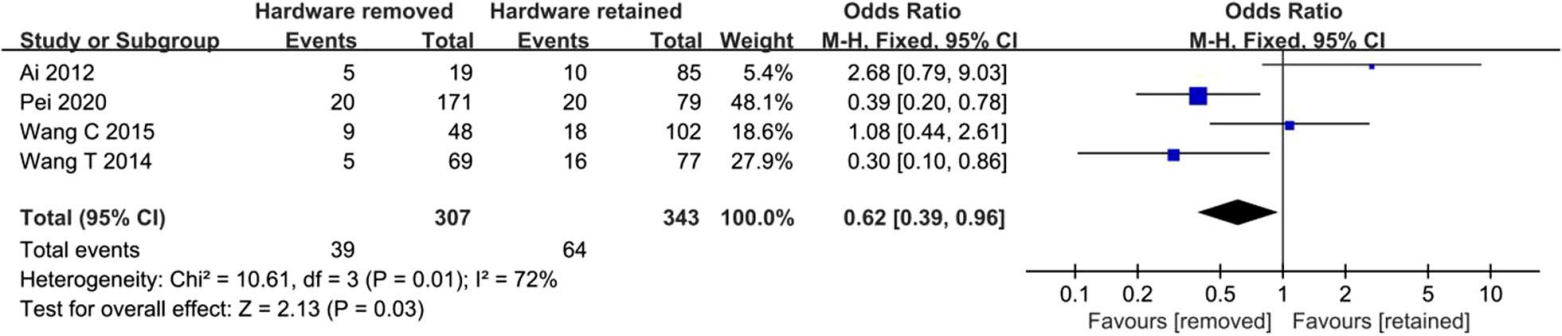
Synthesis of crude odds ratios

**Fig. 3:**
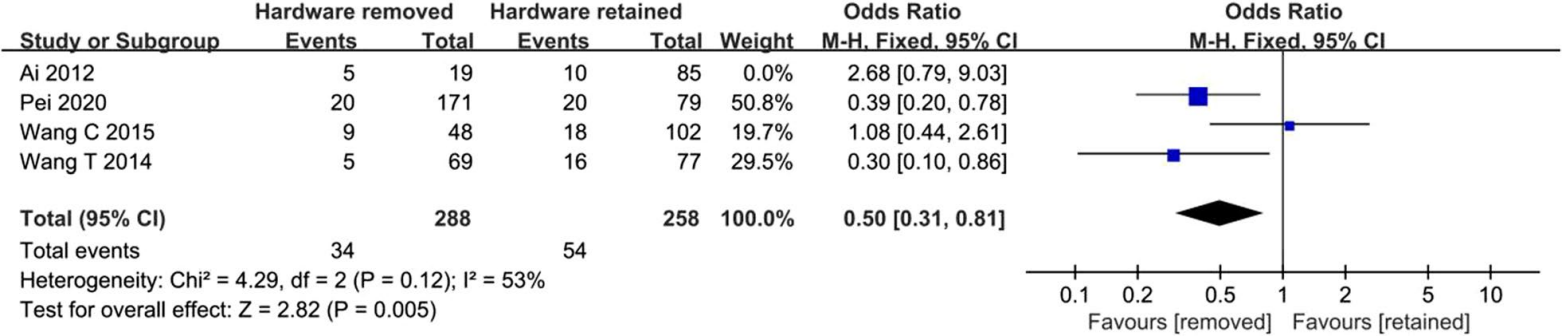
Synthesis of crude odds ratios after sensitivity analysis

Four studies reported events of hardware removal surgery [2, 5, 9, 10]. Lower risk of HR-ONFH was found for hardware removal group compared to hardware retained group (OR 0.62, 95% CI 0.39–0.96; Fig. 2). The statistical significance was not reversed if Ai was left out of the composite (OR 0.50, 95% CI 0.31–0.81; Fig. 3).

### Synthesis of adjusted odds ratios

**Fig. 4:**
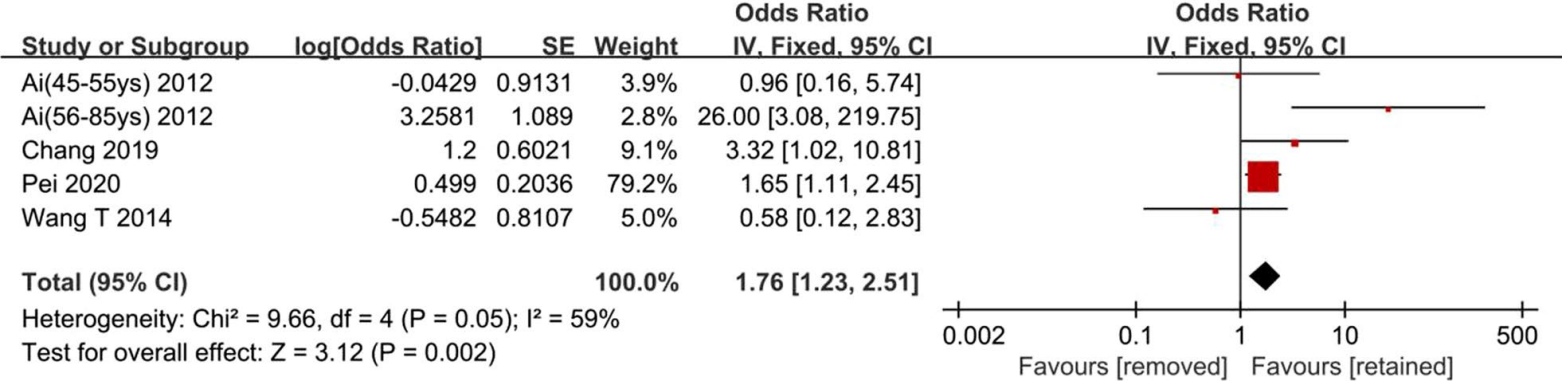
Synthesis of adjusted odds ratios

**Fig. 5:**
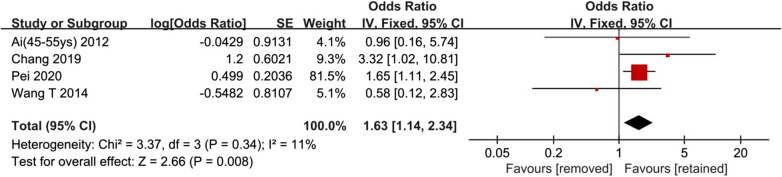
Synthesis of adjusted odds ratios after sensitivity analysis

Four studies investigated the association of hardware status with HR-ONFH [2, 5, 9, 13]. The result of the pooled analysis showed that higher risk of HR-ONFH was associated with hardware removal compared to hardware retained (OR 1.76, 95% CI 1.23–2.51; Fig. 4). Moderate heterogeneity was found across studies. Sensitivity analysis found that the Ai et al. study (subgroup of 56-85 years) was the main source of heterogeneity. I^2^ could decrease from 59 to 11% after this study was removed, and pooled analysis of the remaining studies did not reverse the clinical significance from the original result (OR 1.63, 95% CI 1.14–2.34; Fig. 5), indicating the robustness of the final outcome. A wide range of inclusion criteria may the cause of heterogeneity.

## Discussion

Following internal fixation surgery in patients with femoral neck fractures, ONFH may occur before or after hardware removal. The latter is defined as HR-ONFH by some scholars. This review reveals that it is not an unusual occurrence of HR-ONFH. The pooled rate of HR-ONFH was 12.7% (39/307) in four included studies. In another retrospective study, hidden HR-ONFH was investigated using both simple hip radiography and magnetic resonance imaging (MRI). Incidences of definite ONFH (on X-ray) and hidden ONFH (on MRI) turned out to be 32.1% (18/56) and 23.2% (13/56), respectively. The overall incidence of HR-ONFH even reached 55.4% (31/56) [3]. As most small focal lesions will not progress to femoral head collapse, and simple hip radiography remains the leading screening method at follow-up time, we inter that there may be an underestimate in the diagnosis of HR-ONFH [14].

The association of hardware removal with sequential HR-ONFH has been rarely studied in published literature. The conclusion remains debated regarding whether hardware removal will give rise to the risk of HR-ONFH. In previous studies, the risk of HR-ONFH was assessed mainly using a regression model. This is due to a number of potential predictors of ONFH following FNFs, even with or without internal fixation surgery [2, 5, 9, 10, 13]. In this meta-analysis, the crude ORs exhibited substantial heterogeneity across included studies. This may result from both clinical and methodological differences in studies. The overall effect of crude ORs (OR 0.62, 95% CI 0.39–0.96) favors hardware removal. However, the conclusion is unreliable due to various confounding factors. Definite confounders involve age, gender, fracture classification, reduction quality, time from emergency room to operating room, body mass index (BMI), and American Society of Anesthesiologists scores (ASA) [2, 5, 10]. Significant confounders differ in included studies. Therefore, adjusted ORs were calculated using multivariate logistic regression analysis in four included studies. The overall effect of adjusted ORs (OR 1.76, 95% CI 1.23–2.51) brings out a contrary conclusion to crude ORs, which favors hardware retained. The changed results between crude and adjusted ORs synthesis may be due to changes in included studies and adjustment for potential confounding factors. In the Ai et al. study, patients were divided into two subgroups (45–55 years group and 56–85 years group). In the younger group, no significant association was found between hardware removal and HR-ONFH. Nevertheless, in the elderly group, hardware removal presented with a 26-times higher risk in comparison with the hardware retained group. This study conclusively states that the incidence of HR-ONFH will increase with accrued age of patients [2]. The other three studies did not perform subgroup analysis, even with the age ranging from 18 to 80 years [5, 9, 13]. In sensitivity analysis, the removal of 56–85 years group could decrease statistical heterogeneity, yet the robustness of final conclusion was not diminished. Based on the current meta-analysis, we conclude that hardware removal is associated with an increased incidence of HR-ONFH in patients with femoral neck fractures.

Regarding the pathogenesis of HR-ONFH, several hypotheses have been proposed. Sun et al. raised a theory of micro-fracture [5]. This notion states that femoral neck hardware still bear partial hip stress after fracture healing. Compressive, tensile, and shear stresses are over-concentrated on trabecular bone after hardware removal. Thus, the stress redistribution may lead to micro-fracture around initial screw tunnels, which may further impair the blood supply to femoral head. Another theory is concerning increased intraosseous pressure [15]. Formation of intra-articular hematoma may occur after hardware removal. Subsequently, increased intra-articular pressure may pose a great risk of the femoral head ischemia and secondary ONFH. In addition, hypercoagulability of blood flow may be associated with a thrombus formation, which may reduce the blood supply to femoral head [16]. Kim et al. recently conducted a study identifying hidden ONFH following hardware removal surgery. The investigation using MRI revealed that the lateral pillar of femoral head presents most commonly to encounter ONFH (46.2%) [3]. In another study by Lee et al., lateral pillar lesion involvement was observed in the majority of cases with ONFH (60.6%). This finding is different from the previous opinion that ONFH mainly originates from the central pillar. Furthermore, most hidden HR-ONFH did not progress to collapse during the 2-year follow-up period [14]. This may validate the former hypothesis that the pathogenesis of HR-ONFH differs from traumatic ONFH. Damage to the femoral head blood supply in HR-ONFH patients is not as serious as in traumatic ONFH. Consequently, in younger patients with hardware removal surgery, the reduced blood supply may lead to small focal lesions (i.e., hidden HR-ONFH). As the blood supply to femoral head is more vulnerable in the elderly, collapse is more easily preceded by the slightly impaired blood supply. This situation is in accordance with subgroup analysis in included studies, that accrued age of patients is associated with an increased risk of HR-ONFH [2].

Several clinically significant results have been found in basic patient demographics. In Ai et al. study, compared with younger patients (younger than 55 years of age), hardware removal surgery was more commonly performed in elderly patients (older than 55 years of age) (60/99, 60.6%). Moreover, the primary reason for undergoing hardware removal surgery is religious or superstitious beliefs (63/99, 63.6%) [2]. In other words, the elderly prefer to undergo hardwares removal surgery, not due to postoperative complications (e.g., hardware failure, arthralgia, thigh pain). To the elderly population, hardware removal will pose a higher risk of HR-ONFH, eventuating in undergoing additional revision surgery. From a medical perspective, such consequences could be preventable by renunciation of hardware removal surgery.

In conclusion, in this meta-analysis, hardware removal was associated with an increased incidence of HR-ONFH in patients who underwent internal fixation due to FNF. This information may help surgeons and patients make an informed decision regarding internal hardware removal. However, the present study has certain limitations. First, the strength of this meta-analysis may be reduced by the small number of included studies and sample size. Direct researches on HR-ONFH are rare. Furthermore, the effect size exhibited substantial heterogeneity across studies. Second, included studies were mainly retrospective research with selection bias.

## Data Availability

The present study was a meta-analysis of previous published studies.

## Abbreviations

ONFH: Osteonecrosis of femoral head
FNFs: Femoral neck fractures
HR-ONFH: Hardware removal-induced osteonecrosis of femoral head
RR: Risk rate
OR: Odds ratio
HR: Hazard ratio
